# Novel Nanotherapeutics for Cancer Immunotherapy by PD-L1-Aptamer-Functionalized and Fexofenadine-Loaded Albumin Nanoparticles

**DOI:** 10.3390/molecules28062556

**Published:** 2023-03-11

**Authors:** Xialian Lai, Fengjiao Yao, Yacong An, Xundou Li, Xian-Da Yang

**Affiliations:** Institute of Basic Medical Sciences, Chinese Academy of Medical Sciences & Peking Union Medical College, Beijing 100005, China; laixialian_pumc@126.com (X.L.); fjyao_1103@126.com (F.Y.); anyacong@ibms.pumc.edu.cn (Y.A.); lixd1012@163.com (X.L.)

**Keywords:** PD-L1, aptamer, albumin, nanoparticles, cancer immunotherapy, immune checkpoint, H1-antihistamine, fexofenadine

## Abstract

Immune checkpoint blockade (ICB) is an important strategy for cancer treatment and has achieved remarkable clinical results. Further enhancement of the efficacy of ICB therapy with a new technical approach is of potential medical importance. In this study, we constructed a novel nanotherapeutic agent (PDL1-NP-FEXO) for cancer immunotherapy by attaching PD-L1 aptamers to albumin nanoparticles that were loaded with H1-antihitamine fexofenadine (FEXO). FEXO has been reported to enhance the immunotherapy response by reducing the immunosuppressive M2-like macrophages in the tumor microenvironment. The albumin nanoparticle was fabricated using a self-assembly method. A dynamic light scattering (DLS) study revealed that the average size of PD-L1 aptamer-modified nanoparticle without FEXO (PDL1-NP) was 135.5 nm, while that of PDL1-NP-FEXO was 154.6 nm. Similar to free PD-L1 aptamer, PDL1-NP could also bind with PD-L1-expressing tumor cells (MDA-MB-231). Of note, compared with free PD-L1 aptamer, PDL1-NP significantly boosted tumor inhibition in CT26-bearing mice. Moreover, PDL1-NP-FEXO further enhanced the antitumor efficacy vs. PDL1-NP in an animal model, without raising systemic toxicity. These results indicate that PDL1-NP-FEXO represents a promising strategy to improve ICB efficacy and may have application potential in cancer immunotherapy.

## 1. Introduction

Cancer is a major disease that threatens people’s lives around the world. About 10 million patients died of cancers in 2020, according to the latest *World Cancer Report* published by the WHO. Immune checkpoint blockade (ICB) is a promising antitumor strategy and has opened a new era for cancer therapy. Among ICB therapy, inhibiting the PD-1/PD-L1 pathway is the mainstream approach. PD-1 is expressed on activated T cells, B cells, monocytes, dendritic cells (DCs), and natural killer T cells (NKT) [1]. PD-L1, the ligand of PD-1, is sometimes upregulated in solid tumors [2]. The engagement of PD-L1 with PD-1 induces downregulation of T-cell activity, reduces cytokine production, T-cell lysis, and tolerance to antigens [3], so that tumors can escape the attack by the immune system. Blocking the interaction between PD-1 and PD-L1 theoretically could enhance antitumor immunity and inhibit tumor growth. Indeed, in clinical trials, blockade of the PD-1/PD-L1 pathway has significantly boosted antitumor immunity and prolonged patient survival across multiple tumor types, including colon cancer, melanoma, lung cancer, renal cell carcinoma, ovarian cancer, head and neck cancer, and breast cancer [4,5,6,7,8,9,10]. It is currently well accepted that PD-1/PD-L1 blockade is an important strategy of cancer immunotherapy that deserves further exploration.

Currently, all PD-1/PD-L1 blockers approved for clinical use are antibodies. In addition to antibodies, other types of ligands may also inhibit the PD-1/PD-L1 axis and are under development to become new anticancer immunotherapeutics. Aptamers, for example, may also be used as PD-1/PD-L1 inhibitors. Aptamers are single-strand DNA or RNA with stable tertiary structure that can bind to molecular targets specifically [11]. Compared with antibodies, aptamers have certain technical advantages, including low immunogenicity, better tumor penetration, low production cost, and low batch-to-batch quality variations. Moreover, aptamers can be chemically modified at specific sites for various biomedical applications [12]. Aptamers can also be used as therapeutic agents. Pegaptanib (Macugen^®^) is a VEGF inhibiting aptamer approved by the FDA to treat macular degeneration [13]. Several other aptamers are also in clinical trials and under development [14]. It is currently well recognized that aptamers have good application potential in drug development. Aptamers can also be employed as ICB agent in cancer immunotherapy. Lai et al. developed a high-affinity aptamer for human PD-L1 [15]. Several studies have demonstrated that this aptamer generates good antitumor efficacy in preclinical researches [16,17], indicating that the PD-L1 aptamer has potential for clinical application.

Although ICB has generated remarkable clinical results, the antitumor response is limited in some patients [18]. It is therefore important to further improve the efficacy of ICB therapy with novel therapeutics. It is well recognized that the tumor immune microenvironment (TIME) can influence immunotherapy outcome [19,20,21]. Hence, a combination of ICB therapeutics with TIME-modulating agents theoretically may have the potential to further improve the antitumor response. It has been reported that fexofenadine, a specific H1-antihistamine, could modulate the TIME by reducing the number of immunosuppressive M2-like macrophages and thereby improve T cell antitumor immunity [22]. The M2-like macrophage displays immune suppressive function by expressing high levels of cytokines including IL-10 and TGF-β, thereby promoting tumor growth and affecting the prognosis of cancer patients [23]. It has been reported that patients with higher numbers of M2-like macrophages have a low survival rate [24]. Here, in this study, we designed a novel nanotherapeutic agent (PDL1-NP-FEXO) by attaching PD-L1 aptamers to albumin nanoparticles loaded with FEXO. This approach of co-delivery of PD-L1 aptamer with FEXO in one nanocarrier may have several potential advantages. First, due to the enhanced permeability and retention (EPR) effect, PDL1-NP-FEXO will enrich in tumor tissue, where PD-L1 aptamer and FEXO may produce better antitumor efficacy [25]. Second, FEXO encapsulated in NP has a sustained-release profile, which may extend FEXO lifespan in tumor tissue, thereby influencing the therapeutic outcome. Third, each albumin nanoparticle is functionalized with multiple aptamers, with the potential of multivalent binding to further boost the ICB effect. Fourth, albumin NP has an average size of 130–160 nm that is far above the threshold of renal filtration (about 6 nm) [26,27], while free aptamers are small in size (<5 nm) and easily cleared by renal filtration system [14].

Nowadays, nanomedicine plays an increasingly important role in the field of disease treatment and drug delivery. Since 1995, about 70 nano-drugs have been approved for clinical use by the FDA and EMA [28,29,30,31,32]. Most of these drugs are for cancer therapy. An example of a nano-drug is Abraxane^®^, which is an albumin nanoparticle that carries paclitaxel for the treatment of metastatic breast cancer. These developments indicate that nanomedicine has good clinical translational value and promising application prospects. Numerous nanoparticles have been constructed for drug delivery, in the format of micelles, liposomes, lipid nanoparticles (LNP), and solid nanoparticles. Moreover, the materials used for the construction of these nanostructures are vastly different. Regardless of the material and the format of the nanostructures, nano drug carriers enhanced anticancer efficacy in various tumor models in numerous published animal studies, presumably because of improved drug delivery to tumor tissue due to the EPR effect [33,34]. Here, in this study, we chose an albumin nanoparticle to deliver PD-L1 aptamer and FEXO for several reasons. First, albumin is the most abundant plasma protein and has excellent biocompatibility. Second, recombinant human albumin has been approved by the FDA for clinical use as an excipient. Third, it has been shown that albumin-based nanoparticles have an average size of 100–200 nm, which is appropriate for accumulation in tumor tissue via the EPR effect.

To date, no published study has attempted to develop nanotherapeutics for cancer immunotherapy by co-delivery of PD-L1 aptamer with FEXO in one nanocarrier. We now report that such a nanocomplex could significantly boost the anticancer efficacy of ICB therapy in vivo.

## 2. Results

### 2.1. Conjugation of PD-L1 Aptamer to Albumin

**Figure 1:**
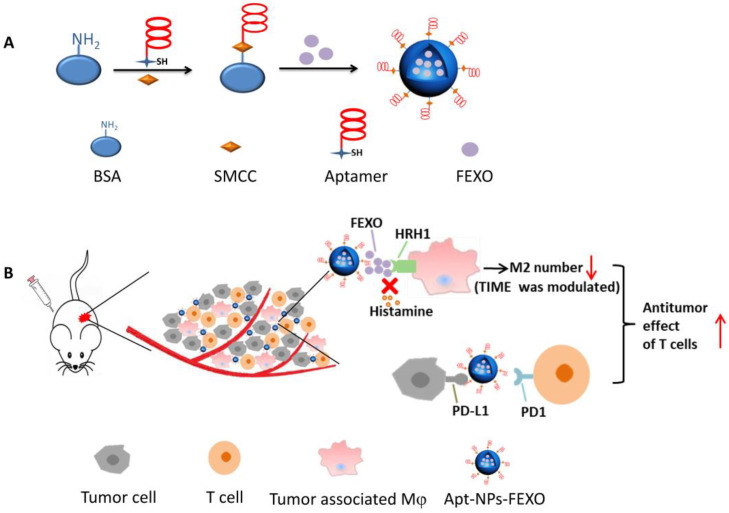
Schematic illustration of the nanostructure designed for cancer immunotherapy. (**A**) Preparation of the PDL1-NP-FEXO. Thiol-modified PD-L1 aptamer was conjugated to the amino-groups of albumin via SMCC. FEXO was encapsulated into aptamer-modified albumin nanoparticle to form the PDL1-NP-FEXO. (**B**) The potential mechanism of PDL1-NP-FEXO for cancer therapy. PDL1-NP-FEXO binds to the PD-L1 expressed on the surface of tumor cells. FEXO blocks the interaction of HRH1 and histamine, reducing number of M2 macrophages in tumor tissue. PD-L1 aptamers on the nanostructure block the PD-1/PD-L1 interaction, boosting the antitumor response of T cells. BSA: bovine serum albumin, SMCC: Sulfosuccinimidyl 4-[N-maleimidomethyl]cyclohexane-1-carboxylate, FEXO: fexofenadine, Mφ: macrophage, HRH1: histamine receptor H1, TIME: tumor immune microenvironment.

**Figure 2:**
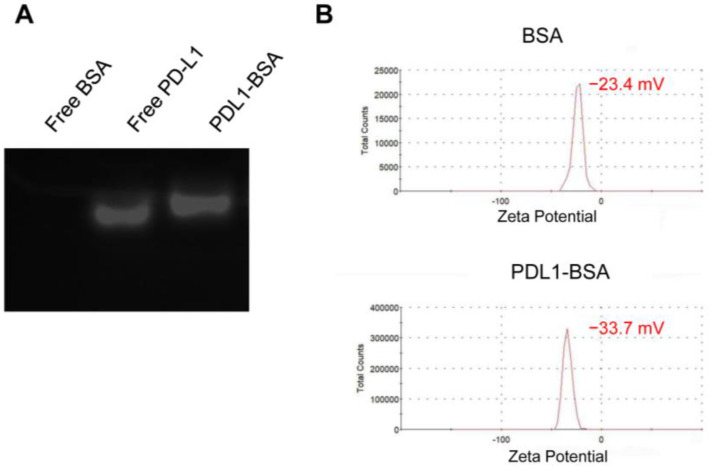
Evaluation of PDL1-BSA conjugation. (**A**) The agarose gel was stained for DNA by GelRed and photographed under ultraviolet light. BSA alone was in Lane 1. Free PD-L1 aptamer was in Lane 2. PDL1-BSA was in Lane 3. (**B**) The zeta potential of BSA (**top**) and PDL1-BSA (**bottom**).

The overall design of the nanostructure was illustrated in Figure 1. Thiol-modified aptamers were conjugated to the amino-groups of albumin (BSA) via Sulfo-SMCC linker to form PDL1-BSA [35]. Aptamer-modified BSA and normal BSA were mixed at a fixed ratio to fabricate the nanoparticle using a previously published method [36]. To evaluate whether the PD-L1 aptamers were linked to BSA, agarose gel electrophoresis of free BSA, free PD-L1 aptamer, and PDL1-BSA was performed. As shown in Figure 2A, free PD-L1 aptamer moved more quickly than PDL1-BSA in the gel (Lanes 2 and 3), suggesting that some aptamers indeed attached to BSA and formed a larger complex. Free BSA generated no signal because the gel was only stained with DNA dyes (Lane 1). To further evaluate the conjugation of aptamer to BSA, we also measured the zeta potentials of plain BSA and PDL1-BSA, which were −23.4 mV and −33.7 mV, respectively (Figure 2B). The fact that PDL1-BSA had more negative charges vs. plain BSA also indicated that the negatively charged DNA aptamers were conjugated to the protein.

### 2.2. Characterization of Nanoparticles

**Figure 3:**
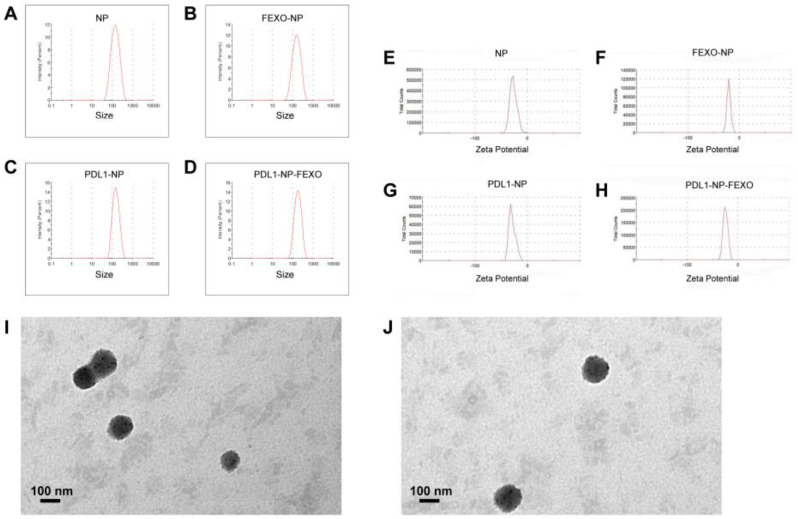
Characterization of the nanoparticles. Size distribution of plain albumin NP (**A**), FEXO-NP (**B**), PDL1-NP (**C**), and PDL1-NP-FEXO (**D**). Zeta potential distribution of plain albumin NP (**E**), FEXO-NP (**F**), PDL1-NP (**G**), and PDL1-NP-FEXO (**H**). TEM images of PDL1-NP (**I**) and PDL1-NP-FEXO (**J**).

For in vivo applications, nanostructures need to be appropriately sized. Nanoparticles smaller than 6 nm tend to be quickly cleared from the circulation by the renal filtration system. However, if particle size exceeds 300 nm, it will be easily sequestrated by the reticuloendothelial system (RES) [37]. The average size of nanoparticles was evaluated by dynamic light scattering (DLS) in this study. As shown in Figure 3 and Table 1, the average sizes of plain albumin NP and PDL1-NP were 117.9 nm and 135.5 nm, respectively (Figure 3A,C), while those of NP-FEXO and PDL1-NP-FEXO were 123.2 nm and 154.6 nm, respectively (Figure 3B,D). The results suggested that all NPs were able to avoid renal leakage and escape RES sequestration. It has been reported that negatively charged nanoparticles tend to stay dispersive and avoid aggregation in blood [38]. We therefore also measured zeta potentials and polydispersity indexes (PDIs) of the nanoparticles. The zeta potentials of plain NP and PDL1-NP were −25.5 mV and −30.7 mV, respectively (Figure 3E,G), while those of NP-FEXO and PDL1-NP-FEXO were −20.8 mV and −24.1 mV, respectively (Figure 3F,H). Of note, compared with NP or NP-FEXO, PDL1-NP or PDL1-NP-FEXO had more negative charges, presumably because of the negatively charged DNA aptamers attached to the nanoparticles. The PDIs of all nanoparticles were less than 0.3, indicating that the sizes of nanoparticles were narrowly distributed.

Transmission electron microscope (TEM) was also employed in this study to characterize nanoparticles. TEM images showed that PDL1-NP-FEXO had a slightly larger size vs. PDL1-NP (Figure 3I,J). Moreover, the drug-loading capacity of NP-FEXO was 1.5 ± 0.06%, and that of PDL1-NP-FEXO was 1.5 ± 0.07% (mean ± SD, *n* = 3). This loading capacity was similar to some of the reported values in the literature, and was sufficient to make NP-FEXO and PDL1-NP-FEXO function in vivo [39]. The encapsulation efficiency of the fexofenadine in NP-FEXO was 29.7 ± 0.6%, and that in PDL1-NP-FEXO was 30.9 ± 0.8% (mean ± SD, *n* = 3).

### 2.3. Release of FEXO from Nanoparticles

**Figure 4:**
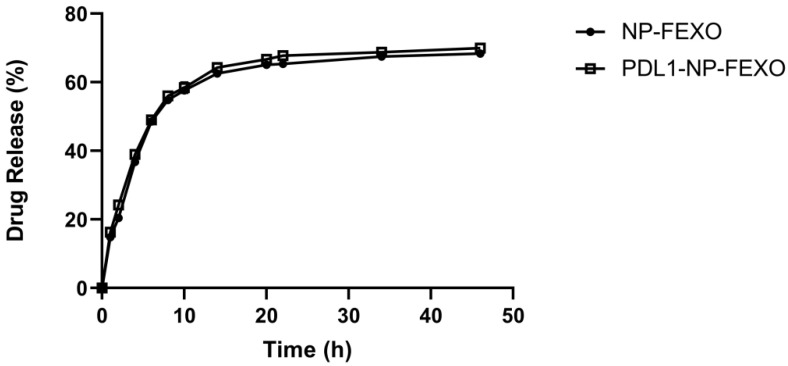
FEXO release kinetics from NP-FEXO or PDL1-NP-FEXO in PBS at pH 7.4 (*n* = 3, mean ± SD).

Sustained release of a drug can prolong a drug’s existing time in the blood or target tissue, thereby influencing its pharmacokinetics and therapeutic efficacy [40]. To determine the FEXO release profile, a dialysis-based drug release assay was performed. The amount of FEXO released at various time points from NP-FEXO or PDL1-NP-FEXO was determined by high-performance liquid chromatography (HPLC). As shown in Figure 4, particle-encapsulated FEXO was released relatively slowly, and the release profiles between PDL1-NP-FEXO and NP-FEXO had no obvious difference. The results indicated that both types of FEXO-loaded nanoparticles exhibited a typical sustained drug release profile.

### 2.4. Affinity of PDL1-NP to PD-L1-Expressing Tumor Cells

**Figure 5:**
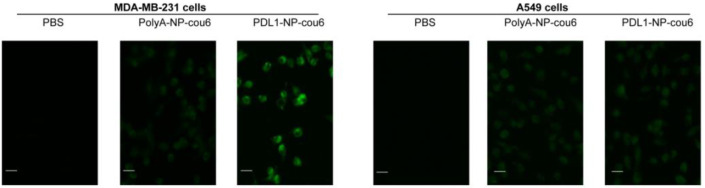
Fluorescent microscopy images of the PD-L1-positive MDA-MB-231 cells (**left**) or PD-L1-negative A549 cells (**right**) treated with PBS, polyA-NP-cou6, or PDL1-NP-cou6. Scale bar represents 30 μm.

PD-L1 aptamer can bind with PD-L1-expressing tumor cells. However, it was unknown whether the PD-L1 aptamers attached to nanoparticles would still enable the particles to bind with PD-L1-expressing tumor cells. To address this issue, a fluorescent dye (Coumarin-6) was encapsulated into aptamer-modified NP (PDL1-NP-cou6) or polyA-modified NP (PolyA-NP-cou6) [41]. PolyA was used here as a control for PD-L1 aptamer, since it could not bind to a target protein specifically due to a lack of complex 3D structures. PD-L1-positive MDA-MB-231 cells and PD-L1-negative A549 cells were incubated with PDL1-NP-cou6 or PolyA-NP-cou6, and evaluated by fluorescence microscopy [42,43,44]. As shown in Figure 5, PD-L1-positive MDA-MB-231 cells treated with PDL1-NP-cou6 generated stronger fluorescent signals compared with those treated with PolyA-NP-cou6, whereas PD-L1-negative A549 cells treated with both nanoparticles generated weak fluorescence (Figure 5). The results suggested that PDL1-NP could preferentially bind with PD-L1-positive tumor cells.

**Figure 6:**
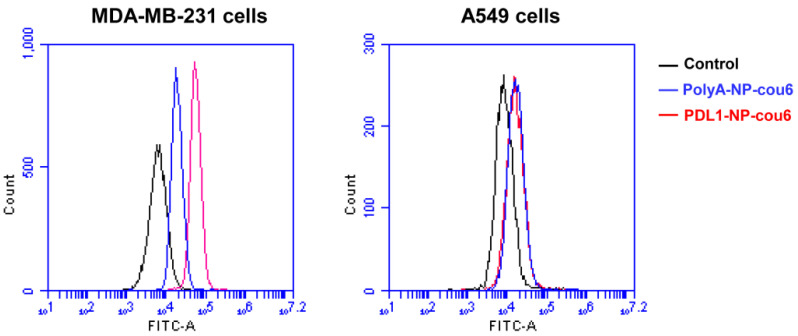
Flow cytometry analysis of PD-L1-positive MDA-MB-231 cells (**left**) or PD-L1-negative A549 cells (**right**) treated with polyA-NP-cou6 (blue) or PDL1-NP-cou6 (red). Signals generated by control cells were in black.

To further validate the results, a flow cytometry study was also conducted. As shown in Figure 6, PD-L1-positive MDA-MB-231 cells treated with PDL1-NP-cou6 generated stronger fluorescent signals vs. those treated by PolyA-NP-cou6, while PD-L1-negative A549 cells treated by PDL1-NP-cou6 generated similar fluorescence as those treated by PolyA-NP-cou6. These data again indicated that PDL1-NP could preferentially bind with PD-L1-expressing tumor cells.

### 2.5. In Vivo Antitumor Study

**Figure 7:**
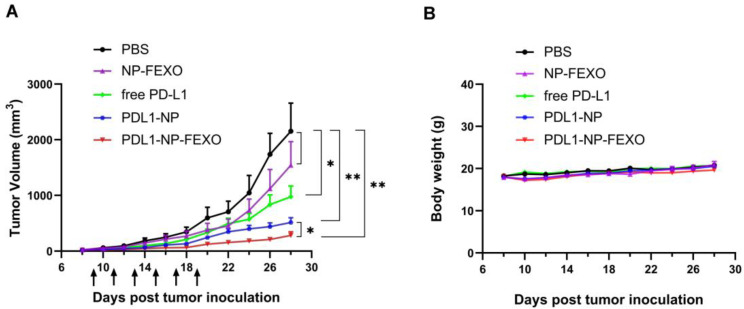
Animal studies in CT26-bearing BALB/c mice. (**A**) Tumor growth curves in mice treated with PBS, free PD-L1 aptamer, PDL1-NP, NP-FEXO, or PDL1-NP-FEXO (*n* = 7, mean ± SEM). (**B**) Body weights of CT26-bearing mice in various treatment groups. The single star (*) indicates statistically significant difference (*p* < 0.05), and the double star (**) indicates statistically significant difference (*p* < 0.01).

Previous research has demonstrated that murine CT26 colon cancer cells expresses PD-L1, and that the PD-L1 aptamer used in this study can bind with CT26 cells and inhibit tumor growth in mice [15]. To investigate whether PDL1-NP-FEXO could boost antitumor efficacy in vivo vs. the free PD-L1 aptamer, CT26 tumor-bearing mice were treated with PBS, free PD-L1 aptamer, PDL1-NP, NP-FEXO, or PDL1-NP-FEXO intraperitoneally every two days for a total of six injections. As shown in Figure 7A, in agreement with previous studies, free PD-L1 aptamers inhibited tumor growth (*p* < 0.05). Of note, PDL1-NP further augmented the antitumor efficacy compared with free PD-L1 aptamer (*p* < 0.05). Importantly, PDL1-NP-FEXO generated the strongest antitumor response, which was even more potent than that generated by PDL1-NP (*p* < 0.05). To investigate whether FEXO was responsible for the strong antitumor effect, NP-FEXO was also administered to the animals. The results showed that NP-FEXO treatment appeared to inhibit tumor growth slightly, but the difference from the control was not statistically significant (*p* = 0.32). To monitor the adverse effects of treatment, body weight was also recorded in this study. There was no obvious difference in body weight among the treatment groups (Figure 7B). These results indicated that PDL1-NP-FEXO could further improve the therapeutic outcome of ICB vs. free PD-L1 aptamer or PDL1-NP, without generating extra systemic toxicity.

## 3. Discussion

Immune checkpoint blockade of the PD-1/PD-L1 pathway has initiated a new era for cancer treatment. In addition to antibodies, aptamers could also act as PD-1/PD-L1 axis inhibitors. Here, in this study, we attempted to improve the antitumor efficacy of a PD-L1 aptamer with nanotechnology. Specifically, the PD-L1 aptamer was attached to an albumin nanoparticle to form a functioned nanocarrier. To modulate the tumor microenvironment, FEXO was also encapsulated in the functional nanoparticle. PDL1-NP and PDL1-NP-FEXO had average diameters of 135.5 nm and 154.6 nm, respectively (Figure 3). Both sizes were above the renal clearance threshold and appropriate for EPR effects. Similar to free PD-L1 aptamer, PDL1-NP could bind with PD-L1-positive tumor cells (Figure 5 and Figure 6). Importantly, the in vivo study showed that PDL1-NP markedly enhanced antitumor efficacy vs. free PD-L1 aptamer (Figure 7). Moreover, PDL1-NP-FEXO further boosted antitumor efficacy compared with PDL1-NP, without raising systemic toxicity. These results indicated that PDL1-NP-FEXO may have better application potential in cancer immunotherapy.

As a novel PD-L1 blocker, aptamer has certain advantages compared with antibody, such as low production cost and better tumor tissue penetration [45]. Nevertheless, free aptamer usually has a small size, resulting in its rapid renal clearance and short circulating time. To address this issue, aptamer was conjugated to macromolecules in previous studies, such as polyethylene glycol (PEG) or dendrimers [46,47]. PEGylation is a commonly used method to prevent renal leakage of aptamer. However, this process may cause some biocompatibility issues, including PEG-related immunogenicity and PEG-containing vacuoles in cells [48]. Dendrimers are macromolecules with hyperbranched defined architectures, and have also been linked to aptamer to reduce its renal leakage. However, cytotoxicity is a concern with currently available dendrimers, and limits the medical application of this approach [49]. At present, it is still necessary to explore other biocompatible and biodegradable molecules for conjugating with aptamers to avoid renal clearance. In this study, the PD-L1 aptamer is conjugated to nanoparticles made of albumin. As the most abundant plasma protein, albumin has low immunogenicity and high biocompatibility [50]. Moreover, recombinant human albumin has been approved by the FDA for clinical use as an excipient. Therefore, PD-L1 aptamer conjugated with albumin nanoparticle has better biocompatibility and clinical application potential.

Indeed, our data showed that PDL1-NP enhanced antitumor efficacy vs. free aptamer (Figure 7). The potential mechanisms of this enhancement may involve several aspects. First, the average size of PDL1-NP is 135.5 nm, which is way above the threshold for renal clearance. Second, the size of PD-L1-NP is appropriate for accumulation in tumor tissue via the EPR effect. EPR is an universal phenomenon in solid tumors, where nanoparticles of a proper size (10–200 nm) can enter the tumor tissue through the vessel wall due to the discontinuity of neo-endothelial cells in tumor blood vessels [51]. Third, each PDL1-NP has multiple aptamers with the potential to generate multivalent binding to PD-L1-positive tumor cells, further boosting the binding affinity and the ICB effect. Taken together, conjugating PD-L1 aptamer to albumin nanoparticle may prolong its circulation time in vivo, enhance tumor-targeting via the EPR effect, and increase targeting affinity by multivalent binding, resulting in improved antitumor efficacy.

In addition to blocking the PD-1/PD-L1 axis, we also want to enhance antitumor efficacy by modulating the tumor immune microenvironment. It has been shown that histamine receptor H1 (HRH1) blockade enhances the therapeutic response of ICB [22]. The possible mechanism is that interdicting the histamine–HRH1 axis reduces the number of immunosuppressive M2-like macrophages, and synergizes with ICB agents to boost the antitumor efficacy of T cells. Here, in this study, a specific H1-antihistamine FEXO was applied together with PDL1-NP to further inhibit tumor growth. Different from previous research, we adopted a new strategy by co-delivering PD-L1 aptamer and FEXO in one nanocarrier. This approach has two potential advantages. On the one hand, FEXO in albumin nanoparticle may enrich tumor tissue due to the EPR effect, reducing the FEXO dosage requirement. On the other hand, FEXO is released from the particle with a sustained release profile, prolonging the pharmacological effects. As shown in Figure 7, the treatment of PDL1-NP-FEXO indeed exhibited stronger antitumor efficacy vs. PD-L1 NP, indicating that codelivery of PD-L1 aptamer and FEXO to tumor tissue could further boost anticancer immunity.

To improve the potential for clinical application, it is desirable that all the components of a therapeutic agent receive prospective regulatory approval. Here, PDL1-NP-FEXO was designed in an effort to meet this requirement. First, albumin is the most abundant plasma protein, so albumin nanoparticles have excellent biocompatibility. Recombinant human albumin has been approved by the FDA for clinical use as an excipient. Second, aptamer also has good biocompatibility and biodegradability. In fact, an aptamer drug (Macugen^®^) was approved for clinical use by the FDA in 2004. Third, the SMCC linker, used here for conjugating albumin with the aptamer, was also approved by the FDA for human use in T-DM1^®^ [52]. Fourth, FEXO is a low-cost antihistamine drug that has been approved by FDA for years. Moreover, the dose of FEXO used in this study is quite low, further reducing the chance of drug-related side effects. Collectively, PDL1-NP-FEXO is made of approvable components and has good potential for clinical application.

Taken together, the therapeutic system developed in this study (PDL1-NP-FEXO) may have certain advantages for cancer immunotherapy. First, the average size of PDL1-NP-FEXO is 154.6 nm, which is much greater than the threshold for renal clearance, increasing the functional lifetime of the PD-L1 aptamer. Second, the PD-L1 aptamer and FEXO carried by albumin nanoparticle may accumulate in tumor tissue due to the EPR effect, enhancing the antitumor effect. Third, FEXO is released from the nanoparticle with a sustained release profile, prolonging the pharmacological effects. Fourth, each PDL1-NP-FEXO has multiple aptamers and may generate multivalent binding to PD-L1-positive tumor cells, further boosting the binding affinity and the ICB effect. However, it should be noted that the current therapeutic system consists of three components, including the PD-L1 aptamer, the fexofenadine, and the albumin NP. In order to realize clinical translation, the PD-L1 aptamer has to be approved for clinical application. Moreover, the procedure for making the aptamer-modified NP should be standardized for large scale production. In addition, extensive studies on pharmacokinetics and pharmacodynamics are also warranted. Nevertheless, the present study suggests that using a nanoparticle to co-deliver PD-L1 aptamer and fexofenadine may achieve better antitumor efficacy and is worthy of further exploration. In conclusion, the co-delivery of PD-L1 aptamer and antihistamine in one nanocarrier significantly enhanced antitumor efficacy in vivo. Our results indicate that PDL1-NP-FEXO represents a promising strategy to improve ICB therapeutic efficacy and may have application potential for cancer immunotherapy.

## 4. Materials and Methods

### 4.1. Cell Lines and Cell Culture

CT26 murine colon carcinoma cells, MDA-MB-231 human breast cancer cells, and A549 human non-small cell lung carcinoma cells were obtained from the Cell Center of the Chinese Academy of Medical Sciences (Beijing, China), and cultured at 37 °C with 5% CO_2_ in DMEM which was supplemented with 100 U/mL penicillin, 100 μg/mL streptomycin, and 10% fetal bovine serum (FBS).

### 4.2. Animals

BALB/c female mice were purchased from Beijing Vital River Laboratory Animal Technology Co., Ltd. (Beijing, China). All mice were fed with a standard diet and water. Mice who were 6–8 weeks old (18–22 g) were selected for experiments. The animal study and procedures were approved by the Ethics Committee of the Institute of Basic Medical Sciences, Chinese Academy of Medical Sciences, according to the institutional animal care and use guidelines.

### 4.3. Reagents

PD-L1 aptamer was modified with a 5′ polyT spacer and 5′thiol. The DNA of SH-TTTTTTTTTT-ACGGGCCACATCAACTCATTGATAGACAATGCGTCCACTGCCCGT was synthesized by Invitrogen (Shanghai, China). Fexofenadine was purchased from Tokyo Chemical Industry Co., Ltd. (Shanghai, China). Bovine serum albumin (BSA) was purchased from TBD Science Bio-engineering Co., Ltd. (Tianjin, China). Tris (2-carboxyethyl) phosphine (TCEP) and coumarin 6 were purchased from Sigma-Aldrich (Shanghai, China). Sulfosuccinimidyl 4-[N-maleimidomethyl]cyclohexane-1-carboxylate (Sulfo-SMCC) was purchased from Jinsui Bio-Technology Co., Ltd. (Shanghai, China).

### 4.4. Conjugation of Aptamer to Albumin

For preparation of PDL1-BSA, thiol-modified PD-L1 aptamer was conjugated to the amino groups of albumin via a standard linker, Sulfo-SMCC [35,53]. Then, 4 mg BSA and 0.72 mg Sulfo-SMCC were reacted in 950 μL PBS (pH 7.2) for 3 h and ultrafiltrated with a 30 kD cut-off tube to remove unreacted Sulfo-SMCC. SMCC-modified BSA was resuspended in 200 μL PBS. The protein concentration of SMCC-modified BSA was measured by standard BCA assay. Equimolar thiol-modified PD-L1 aptamer was dissolved in 190 μL Milli-Q water, which was mixed with 10 μL 800 mM TCEP solution and reacted for 1 h. Next, the aptamer solution was mixed with the SMCC-modified BSA solution, and reacted overnight. The product was ultrafiltrated with a device of 30 KD cut-off, and suspended in 20% sodium chloride or 10 × PBS. The protein and DNA concentrations of the final mixture were measured per standard protocol.

### 4.5. Preparation of Aptamer-Modified Nanoparticles

PDL1-NP-FEXO was made using a modified self-assembling method [54,55]. Briefly, 1.3 mL 20% sodium chloride was mixed with 1 mL pure ethanol containing 1 mg FEXO. Next, this solution was mixed with 0.1 mL 20% sodium chloride containing 19.6 mg BSA and 0.4 mg PDL1-BSA monomer, incubated at 65 °C for 10 min. The mixture was put onto a rotating mixer and mixed at 40 rpm for 20 min at room temperature. Next, the mixture was poured into 3.5 mL Milli-Q water and heated at 65 °C under rapid stirring for 20 min. The solution was put into an ice bath for 10 min. The preparation of NP-FEXO was similar to that of PDL1-NP-FEXO, except that BSA was used in place of PDL1-BSA.

PDL1-NP was prepared using a similar procedure. Briefly, 0.65 mL 9% sodium chloride was mixed with 1 mL pure ethanol solution. This solution was mixed with 0.1 mL 9% sodium chloride containing 19.6 mg BSA and 0.4 mg PDL1-BSA monomer, and incubated at 65 °C for 10 min. The mixture was put on a rotating mixer and mixed at 40 rpm for 20 min at room temperature. Next, the mixture was mixed with 10.5 mL Milli-Q water, which was heated at 65 °C under rapid stirring for 20 min. The mixture was put into an ice bath for 10 min. The preparation of NP was similar to that of PDL1-NP, except that plain BSA was used in place of PDL1-BSA.

PDL1-NP-cou6 was prepared using a protocol similar to when preparing PDL1-NP, except that coumarin6 ethanol solution (0.0078 mg/mL) was used in place of pure ethanol. PolyA-NP-cou6 was prepared similarly, except that polyA-BSA was used in place of PDL1-BSA.

### 4.6. Assessment of DNA Conjugation to Albumin

Agarose gel electrophoresis was applied to evaluate whether PD-L1 aptamer was conjugated to albumin. One percent (*w*/*v*) agarose gel containing the GelRed DNA dye (Invitrogen, Shanghai, China) was prepared with 0.5× Tris-Borate-EDTA (TBE) buffer. To minimize the influence of albumin’s electrical charge on the movement, the pH of TBE buffer was adjusted to approximately 4.8, which was close to the isoelectric point of albumin. Free albumin, free PD-L1 aptamer, and PDL1-BSA were loaded into the gel, and subjected to 90 V for 8 min. The DNA was visualized by exposing the gel under UV light by an UV documentation device (Alliance, London, UK).

### 4.7. Characterization of Nanoparticles

The average particle size distribution, polydispersity index (PDI), and zeta potential of nanoparticles were determined at 25 °C by DLS using Zeta Sizer Nano ZS90 (Malvern Instruments, Malvern, UK).

The morphology of nanoparticles was observed by transmission electron microscope (TEM) (JEM-200CX, JEOL, Tokyo, Japan) after the samples were deposited on a copper grid and stained with 3% phosphotungstic acid.

### 4.8. Measurement of Drug-Loading Capacity and Drug Encapsulation Efficiency

For evaluation of the percentage of fexofenadine entrapped in NPs, 1 mL of drug-loaded samples was filtered with an ultrafiltration device. The filtrate was determined by high-performance liquid chromatography (HPLC) with a UV-Vis detector at 220 nm, using a C18 column (Diamonsil C18 (2), 5 μm 100 Å 250 × 4.6 mm) [56]. The flow rate was 1.0 mL/min, and the column temperature was 40℃. The mobile phase was composed of acetonitrile and 0.1% phosphoric acid. The volume ratio of acetonitrile to 0.1% phosphoric acid was 20:80 at first, and gradually turned to 70:30 within 20 min, and was maintained at 70:30 for 10 min. The calibration curve was linear in the range of 0.78–200 μg/mL, with a correlation coefficient of R^2^ = 0.994. The drug-loading capacity (DL) and drug encapsulation efficiency (EE) were calculated by the following equations:

$$\mathrm{DL}\%=\frac{\mathrm{total}\;\mathrm{FEXO}\;\mathrm{added}\;\mathrm{in}\;\mathrm{NPs}\;-\;\mathrm{unencapsulated}\;\mathrm{FEXO}}{\mathrm{total}\;\mathrm{nanoparticles}}\times 100\%$$

$$\mathrm{EE}\%=\frac{\mathrm{total}\;\mathrm{FEXO}\;\mathrm{added}\;\mathrm{in}\;\mathrm{NPs}\;-\;\mathrm{unencapsulated}\;\mathrm{FEXO}}{\mathrm{total}\;\mathrm{FEXO}\;\mathrm{added}}\times 100\%$$

### 4.9. Drug Release Study

To evaluate drug release, 3 mL of drug-loaded samples was filtered with an ultrafiltration device and resuspended with the same volume of PBS. The suspension was put into a bag made of dialysis membrane (8000–14,000, Solarbio, Beijing, China). The dialysis bag was immersed in 45 mL of PBS (pH 7.4) at 37 °C and put on a shaker that shook at 200 rpm. At indicated time points, 1 mL buffer was withdrawn and replaced with an equal volume of fresh PBS. The amount of drug released at each time point was determined by high-performance liquid chromatography (HPLC) described above. The cumulative drug release rate (Q%) was calculated using the following equation [57]:

$$\mathrm{Cumulative}\;\mathrm{drug}\;\mathrm{release}\;(100\%)=\frac{\mathrm{FEXO}\;\mathrm{released}\;\mathrm{at}\;\mathrm{time}}{\mathrm{encapsulated}\;\mathrm{FEXO}\;\mathrm{in}\;\mathrm{nanoparticles}}\times 100\%$$

### 4.10. Affinity of PDL1-NP to PD-L1-Expressing Tumor Cells

To determine whether PDL1-NP could still bind to PD-L1-expressing cells, MDA-MB-231 cells or A549 cells (1 × 10^4^) were cultured onto 96-well plates. After 12 h, cells were treated with PBS, polyA-NP-cou6, or PDL1-NP-cou6 (cou6 concentration of 0.06 μg/mL) for 30 min. The cells were washed thrice with PBS, and imaged using an inverted fluorescent microscope (Leica DMi8, Wetzlar, Germany).

Flow cytometry can also be performed to evaluate the binding of PDL1-NP with PD-L1-expressing cells. MDA-MB-231 cells or A549 cells (2 × 10^5^) were treated with PBS, polyA-NP-cou6, or PDL1-NP-cou6 (cou6 concentration: 0.06 μg/mL) for 30 min. Afterwards, cell medium was removed, and the cells were washed with PBS thrice. Cellular fluorescent signals were determined by Accuri C6 plus Flow Cytometer (BD Biosciences, San Jose, CA, USA).

### 4.11. Animal Study

BALB/c mice who were 6–8 weeks old with an average weight of 20 g were used for the animal study. CT26 cells (2 × 10^5^) were suspended in 100 μL PBS and injected subcutaneously into the right rear flank of mice [58,59]. When tumors were palpable, mice were randomly divided into various treatment groups (7 animals per group). The mice were treated with PBS, free PD-L1 aptamer, PDL1-NP, PDL1-NP-FEXO, or FEXO-NP via intraperitoneal injection every two days for a total of six injections. The dosages of PD-L1 aptamer for free aptamer, PDL1-NP, and PDL1-NP-FEXO groups were 1.2 mg/kg per animal in 200 μL PBS. Animals in the NP-FEXO group received the same amount of FEXO (5 mg/kg) as the PDL1-NP-FEXO group. Tumor sizes and body weights were measured every 2 days during the experiment. Tumor volume was calculated according to the formula (a × b^2^)/2, where a and b represent the length and width of the tumor, respectively.

### 4.12. Statistical Analysis

Statistical analysis was performed by GraphPad Prism 8 software (La Jolla, CA, USA). Two-tailed Student’s *t*-test was used for statistical calculation. A *p* value of less than 0.05 was regarded as statistically significant.

## Figures and Tables

**Table 1:** Size and Zeta Potential of Nanoparticles (*n* = 3, mean ± SD).

Formulation	Size (nm)	PDI	Zeta Potential (mV)
NP	117.9 ± 2.7	0.254 ± 0.018	−25.5 ± 1.04
PDL1-NP	135.5 ± 1.95	0.234 ± 0.031	−30.7 ± 1.32
NP-FEXO	123.2 ± 3.39	0.247 ± 0.024	−20.8 ± 0.53
PDL1-NP-FEXO	154.6 ± 2.92	0.222 ± 0.011	−24.1 ± 0.72

## Data Availability

The data presented in this study are available in this article.

## Sample Availability

Samples of the compounds are not available from the authors.
